# Maintenance Therapy After Allogeneic Stem Cell Transplantation in Acute Myeloid Leukemia: Still an Open Question?

**DOI:** 10.3390/cancers18213487

**Published:** 2026-10-29

**Authors:** Alessandra Sperotto, Federico De Marchi, Marco Basso, Giulia Ciotti, Alessandro Atanasio, Roberta De Marchi, Michele Gottardi

**Affiliations:** 1 UOSD Terapie Cellulari, Dipartimento di Oncologia, Istituto Oncologico Veneto (IOV), Istituto di Ricovero e Cura a Carattere Scientifico (IRCCS), 35128 Padua, Italygiulia.ciotti@iov.veneto.it (G.C.); alessandro.atanasio@iov.veneto.it (A.A.); roberta.demarchi@iov.veneto.it (R.D.M.); michele.gottardi@iov.veneto.it (M.G.); 2 UOC Oncoematolgia, Dipartimento di Oncologia, Istituto Oncologico Veneto (IOV), Istituto di Ricovero e Cura a Carattere Scientifico (IRCCS), 35128 Padua, Italy; 3 UOC Farmacia, Dipartimento di Oncologia Traslazionale e dei Servizi Trasversali, Istituto Oncologico Veneto (IOV), Istituto di Ricovero e Cura a Carattere Scientifico (IRCCS), 35128 Padua, Italy; marco.basso@iov.veneto.it

**Keywords:** acute myeloid leukemia, allogeneic stem cell transplantation, maintenance therapy, targeted therapy

## Abstract

Disease relapse remains the leading cause of treatment failure in patients with acute myeloid leukemia (AML) undergoing allogeneic hematopoietic cell transplantation (allo-HCT). This narrative review, searched from Pubmed, from 2015 to 2026, critically synthesizes the available evidence on post-transplant maintenance strategies, which have evolved over the past three decades from conventional chemotherapy to molecularly targeted agents and interventions aimed at modulating antileukemic immunity. Particular attention is given to the biological rationale, efficacy, safety, and clinical applicability of these approaches, as well as to the substantial heterogeneity in study design, patient selection, and treatment initiation and duration. The available evidence indicates that the potential benefit of maintenance is unlikely to be uniform across all recipients and should be interpreted in relation to AML molecular features, baseline relapse risk, prior treatment exposure, measurable residual disease status, expected toxicity, and the patient’s overall clinical condition. Although selected strategies may prolong remission and improve survival, the optimal agent, timing, duration, and target population remain incompletely defined. Current evidence therefore supports a risk-adapted and molecularly informed approach, while underscoring the need for adequately powered prospective studies using standardized endpoints and incorporating quality-of-life outcomes.

## Simple Summary

Relapse remains the main cause of treatment failure after allogeneic stem cell transplantation for acute myeloid leukemia. Maintenance therapy aims to control residual leukemia while the donor immune system recovers, but its benefits and risks differ among patients and treatments. This review summarizes current evidence for targeted drugs, including FLT3 and IDH inhibitors, hypomethylating agents, combination regimens, and emerging therapies. The strongest evidence supports sorafenib for selected patients with FLT3-ITD-mutated leukemia, while gilteritinib may be most useful when highly sensitive testing detects residual FLT3-ITD before or after transplantation. Other approaches remain promising but are supported mainly by small or early-phase studies, and treatment can be limited by low blood counts, infections, graft-versus-host disease, drug interactions, and incomplete treatment exposure. Overall, maintenance should not be offered uniformly to every transplant recipient. Decisions should consider leukemia genetics, relapse risk, measurable residual disease, previous therapy, recovery after transplantation, tolerability, and competing complications. Well-designed prospective trials are needed to define which patients benefit, when treatment should begin, the most appropriate dose and duration, and whether fixed maintenance or measurable residual disease-guided pre-emptive therapy provides the best balance between relapse prevention, quality of life, and safety.

## 1. Introduction

Over the past decade, advances in the molecular characterization of acute myeloid leukemia (AML) have profoundly reshaped its biological interpretation and clinical management. The widespread implementation of next-generation sequencing (NGS) has moved AML classification beyond traditional morphologic and cytogenetic criteria toward a more refined, mutation-informed framework, thereby improving prognostic stratification and supporting the development of genomically informed therapeutic strategies [1]. Molecular profiling has also strengthened the role of measurable residual disease (MRD) assessment, which increasingly serves as a dynamic marker of treatment response and relapse risk [2]. Within this evolving framework, several new therapeutic approaches have entered clinical practice: targeted ones (including FLT3, IDH1/2, BCL-2 and Hedgehog pathway inhibitors), gemtuzumab ozogamicin (GO), and new formulations of older drugs (liposomal daunorubicin/cytarabine (CPX-351) in selected settings [1,2]. More recently, menin inhibitors have shown encouraging activity in KMT2A/MLL-rearranged or NPM1-mutated AML, with responses in the relapsed/refractory setting varying by agent, molecular subgroup and response definition [3,4].

These developments have substantially expanded the therapeutic landscape and contributed to higher remission rates in selected settings, but they have not eliminated the central challenge of durable disease control. Even MRD negativity does not invariably ensure long-term remission, and relapse remains frequent, particularly in higher-risk disease patients who do not proceed to allogeneic transplant-based consolidation. Thus, the benefit of molecularly guided therapy remains limited by clonal heterogeneity, adaptive resistance, and variable response durability, underscoring the persistent gap between increasingly sophisticated disease classification and potentially curative treatment strategies [5,6,7].

This gap is particularly evident in patients without therapeutically actionable mutations, for whom treatment remains largely based on conventional risk-adapted strategies. Intensive chemotherapy is still the standard first-line approach for patients considered suitable for intensive treatment, but its curative potential is limited by intrinsic disease biology, early chemoresistance and treatment-related toxicity. Lower-intensity regimens provide an important alternative for patients considered unsuitable for intensive chemotherapy, although they often represent a compromise between tolerability and antileukemic efficacy rather than an equivalent curative option.

Against this background, allogeneic hematopoietic stem cell transplantation (allo-HCT) retains a central role because it combines cytoreduction with graft-versus-leukemia activity and remains the only potentially curative strategy for many patients with AML. However, its benefit is not uniform and depends on an individualized balance among relapse risk, donor availability, patient fitness, disease biology, treatment response and transplant-related morbidity and mortality.

This balance has become increasingly relevant as refinements in conditioning, graft-versus-host disease (GVHD) prophylaxis, anti-infective strategies and supportive care have broadened eligibility to older patients and selected individuals with comorbidities. Yet improved feasibility should be interpreted cautiously, as lower transplant-related mortality is strongly influenced by patient selection, transplant timing, donor characteristics and center-specific expertise, and may be offset by competing non-relapse risks.

Even with these improvements, post-transplant relapse remains the major limitation of allo-HCT, illustrating the unresolved discrepancy between transplant feasibility and durable disease eradication [1]. Reported relapse rates of approximately 30% to 70% vary according to disease risk, remission status, MRD burden, conditioning intensity, donor source and post-transplant immune dynamics [1]. As transplantation is increasingly applied across broader AML populations, relapse continues to constrain long-term survival and supports the need for risk-adapted, biologically informed post-transplant interventions [8,9].

The rationale for such interventions is grounded in the biology of relapse, which reflects a dynamic interaction between residual leukemic burden and recovering donor immunity. Leukemic clones may survive conditioning, persist during immune equilibrium and subsequently evade graft-versus-leukemia surveillance through clonal selection, immune editing or rapid expansion before full donor immune reconstitution [1,2,8,9]. This dynamic process limits the utility of static pre-transplant risk models alone for post-transplant decision-making.

Accordingly, maintenance and pre-emptive strategies after allo-HCT have been proposed to address residual disease during the vulnerable phase of immune reconstitution [10,11]. Targeted agents may suppress residual leukemic clones, enhance susceptibility to graft-versus-leukemia effects, or merely delay overt relapse without modifying immune escape. Their timing, duration, patient selection and effects on the balance between graft-versus-host disease and graft-versus-leukemia activity remain uncertain, particularly during immune reconstitution. Maintenance should therefore be developed as a risk-adapted and MRD-informed intervention rather than applied uniformly to all transplanted patients [11,12,13,14].

For FLT3-ITD-mutated AML, current ELN-DAVID guidance recommends gilteritinib maintenance when FLT3-ITD MRD is detected before or after allo-HCT by an ultrasensitive molecular assay [15].

This review critically examines current and emerging maintenance strategies after allo-HCT in AML, focusing on biological rationale, strength of evidence, clinical applicability and unresolved limitations. It evaluates how relapse risk, MRD dynamics, molecular biomarkers, tolerability and graft-versus-leukemia interactions may guide patient selection, with the aim of distinguishing strategies supported by sufficient biological and clinical justification from those that remain insufficiently supported for routine use (Figure 1).

## 2. Risk Stratification and Predictors of Relapse After allo-HCT

Because maintenance strategies are unlikely to benefit all transplanted patients equally, their rational implementation requires accurate identification of those at greatest risk of relapse [6,16,17,18,19]. The identification—in the pre-transplant phase—of prognostic factors associated with a high risk of post-transplant relapse, followed by risk-based patient stratification, may improve the rational use of post-transplant maintenance therapy [1,18].

Such stratification is increasingly understood as a dynamic process rather than a single pre-transplant assessment [18]. For each patient with AML, disease risk can be conceptualized as both “static” and “dynamic”. Static risk is determined at diagnosis by cytogenetic abnormalities and molecular biology, as captured by ELN 2022 risk categories, developed primarily for intensively treated AML, and contributes to the initial assessment of allo-HCT eligibility [16]. A separate ELN 2024 genetic risk framework addresses patients receiving less-intensive therapies [20]. Dynamic risk, by contrast, is defined by the response to induction and subsequent therapy, including primary resistance, relapse, depth of remission and evolving measurable residual disease (MRD) status [6,17,19]. Accordingly, even patients initially classified as having favorable cytogenetic or molecular risk may become candidates for allo-HCT if resistance or relapse emerges during first-line therapy. Moreover, static risk should be reassessed at relapse, as clonal evolution and newly detectable mutations, particularly those identified by next-generation sequencing, may alter prognosis and therapeutic strategy [16,18] (Figure 2).

## 3. Pharmacologic Maintenance Strategies After allo-HCT

On this basis, pharmacologic maintenance strategies can be organized according to their biological rationale, targetable molecular lesion and strength of clinical evidence. Among mutation-directed approaches, FLT3 inhibition represents the most extensively studied paradigm.

### 3.1. Targeted Therapy

#### 3.1.1. FLT3 Inhibitors

FLT3-ITD mutations occur in approximately 25% of newly diagnosed AML and have historically been associated with an adverse prognosis, largely because of an increased risk of early relapse. The incorporation of FLT3 inhibitors into intensive chemotherapy and the routine use of allo-HCT as consolidation in eligible patients have improved outcomes in this molecular subgroup. Nevertheless, relapse after transplantation remains clinically relevant, with reported rates of approximately 30–40% [21].

The availability of several FLT3 inhibitors, together with the frequent persistence of FLT3-ITD at relapse after transplantation, has provided a strong rationale for post-transplant FLT3-directed maintenance. Nevertheless, the evidence base is uneven across agents and is influenced by heterogeneous trial designs, variable timing of treatment initiation, differences in MRD assessment, and incomplete information on the optimal duration of maintenance. Among available agents, sorafenib has generated the most mature and consistent post-allo-HCT data, whereas the roles of gilteritinib, quizartinib and midostaurin remain more context-dependent and require more cautious interpretation.

Brunner A. et al. evaluated sorafenib maintenance after allo-HCT in a retrospective study of adults with FLT3-ITD-mutated AML transplanted in first complete remission (CR1) [22]. Between 2008 and 2014, 26 patients who received sorafenib were compared with 55 matched controls. Sorafenib was initiated at a median of 68 days after transplantation and was analyzed as a time-varying covariate in overall survival (OS) and progression-free survival (PFS) analyses; a landmark analysis was also performed including controls who were alive and relapse-free at the median time of sorafenib initiation. Median follow-up was 27.2 months after transplant among sorafenib-treated survivors and 38.4 months among controls (*p* = 0.021). In the landmark analysis, sorafenib maintenance was associated with improved 2-year OS compared with controls (81% vs. 62%, *p* = 0.029), as well as superior 2-year PFS (82% vs. 53%, *p* = 0.0081) and a lower 2-year cumulative incidence of relapse (8.2% vs. 37.7%, *p* = 0.0077). In multivariable analysis, sorafenib remained significantly associated with improved OS (hazard ratio [HR] 0.26, *p* = 0.021) and PFS (HR 0.25, *p* = 0.016). No significant differences were observed in 2-year non-relapse mortality (9.8% vs. 9.3%, *p* = 0.82) or 1-year chronic graft-versus-host disease (cGVHD) (55.5% vs. 37.2%, *p* = 0.28). Although these findings suggested that post-transplant sorafenib maintenance may reduce relapse risk and improve survival in patients with FLT3-ITD-mutated AML, the retrospective design, limited sample size and potential selection bias restrict the strength of the conclusions. The study therefore provided an important hypothesis-generating signal and a rationale for prospective testing, rather than definitive proof of benefit [22].

Chen YB et al. evaluated sorafenib maintenance after allo-HCT in a phase I study (ClinicalTrials.gov NCT01398501) [23]. The trial followed a 3 + 3 dose-escalation design, with additional patients treated at the maximum tolerated dose (MTD). Overall, 22 patients with different disease status at transplant were enrolled. Sorafenib was initiated between day 45 and day 120 after transplant and administered for up to twelve 28-day cycles. The MTD was 400 mg twice daily: only one dose-limiting toxicity (DLT), a pericardial effusion, was reported. Two patients discontinued treatment because of relapse, and five stopped sorafenib because of drug-related toxicities occurring after the DLT assessment period. After a median follow-up of 16.7 months from HCT among surviving patients (range 8.1–35.0), only one case of grade 2 GVHD occurred, whereas the 12-month cumulative incidence of chronic GVHD was 38% (90% confidence interval [CI], 21–56%). In the overall cohort, 1-year progression-free survival (PFS) from transplantation was 85% (90% CI, 66–94%) and 1-year overall survival (OS) was 95% (90% CI, 79–99%). Among patients transplanted in CR1 and CR2 (*n* = 19), 1-year PFS was 95% (90% CI, 76–99%) and 1-year OS was 100%, with only one relapse. These results supported feasibility and informed dose selection, but the single-arm design, small sample size, and enrichment for patients able to start maintenance after early post-transplant recovery limit any inference regarding efficacy [23].

The randomized, placebo-controlled phase II SORMAIN (SORafenib MAINtenance) trial (German Clinical Trials Register: DRKS00000591) evaluated post-transplant sorafenib maintenance in patients with FLT3-ITD-mutated AML who were in complete hematologic remission after allo-HCT [24]. Patients were assigned to receive sorafenib (*n* = 43) or placebo (*n* = 40) for up to 24 months, with relapse-free survival (RFS) as the primary endpoint. Although accrual was limited, with 83 patients enrolled over 5 years, the study demonstrated a clinically meaningful benefit in favor of sorafenib. At a median follow-up of 41.8 months, sorafenib significantly reduced the risk of relapse or death compared with placebo (hazard ratio [HR], 0.39; 95% confidence interval [CI], 0.18–0.85; log-rank *p* = 0.013). The 24-month RFS probability was 85.0% in the sorafenib arm versus 53.3% in the placebo arm (HR, 0.256; 95% CI, 0.10–0.65; log-rank *p* = 0.002). Exploratory analyses suggested that the greatest benefit was observed among patients with undetectable MRD before transplantation and among those with detectable MRD after transplantation. Despite the small sample size and slow accrual, SORMAIN remains a key study because of its placebo-controlled design and durable follow-up; however, its findings still leave unresolved questions regarding patient selection, treatment duration, and interaction with MRD status and immune-mediated graft-versus-leukemia effects [24].

Also, Xuan L et al. [25] reported the results of a randomized phase III trial evaluating sorafenib as post-transplant maintenance in patients with FLT3-ITD-mutated AML (NCT02474290). Eligible patients were 18–60 years of age, were in composite complete remission before and after transplantation, and had achieved hematopoietic recovery within 60 days after allo-HCT. Patients were randomly assigned in a 1:1 ratio to receive sorafenib maintenance at a dose of 400 mg orally twice daily, initiated on days 30–60 and continued to day 180 after transplant, or no maintenance therapy. The primary endpoint was the 1-year cumulative incidence of relapse in the intention-to-treat population [25].

Between 20 June 2015, and 21 July 2018, 202 patients were enrolled (sorafenib maintenance *n* = 100; no maintenance *n* = 102). After a median post-transplant follow-up of 21.3 months (interquartile range [IQR], 15.0–37.0), sorafenib maintenance significantly reduced the 1-year cumulative incidence of relapse compared with the control group (7.0%; 95% CI, 3.1–13.1 vs. 24.5%; 95% CI, 16.6–33.2; hazard ratio [HR], 0.25; 95% CI, 0.11–0.57; *p* = 0.0010). The most frequent grade 3–4 adverse events were infections (25% in the sorafenib group vs. 24% in the control group), acute GVHD (23% vs. 21%), chronic GVHD (18% vs. 17%), and hematologic toxicity (15% vs. 7%). No treatment-related deaths were reported [25]. Long-term follow-up confirmed sustained benefit: 5-year OS was 72.0% vs. 55.9% (HR, 0.55; 95% CI, 0.34–0.88; *p* = 0.011), leukemia-free-survival was 70.0% vs. 49.0% (HR, 0.47; 95% CI, 0.30–0.83; *p* = 0.0007), and cumulative relapse incidence was 15.0% vs. 36.3% (HR, 0.33; 95%CI, 0.18–0.60; *p* = 0.0003) [26].

These randomized data provide the strongest support for sorafenib as post-allo-HCT maintenance; however, interpretation should consider the open-label design, the focus on early relapse as the primary endpoint, and the restricted eligibility of patients who achieved hematologic recovery within 60 days after transplantation. Pre-transplant sorafenib exposure was common (59.0% vs. 56.0%), and 59.0% of maintenance recipients required dose modification [25,26]. Thus, while sorafenib has the most compelling evidence among FLT3 inhibitors, its routine use still requires individualized assessment of tolerability, GVHD risk, drug interactions and duration of therapy.

The Blood and Marrow Transplant Clinical Trials Network evaluated gilteritinib maintenance after allo-HCT in MORPHO (NCT02997202), an international, multicenter, phase III, double-blind, randomized, placebo-controlled trial. Patients in CR1 after transplantation were assigned to gilteritinib 120 mg once daily or placebo for up to 24 months, with RFS as the primary endpoint. Overall, 356 patients were randomized. Although the trial did not meet its primary endpoint in the overall population, because the improvement in RFS was not statistically significant (HR, 0.679; 95% CI, 0.459–1.005; *p* = 0.0518), clinically relevant differences emerged according to MRD status [27,28].

In the prespecified subgroup of patients with detectable MRD before and/or after transplantation, assessed using a highly sensitive PCR-NGS FLT3-ITD MRD assay, gilteritinib maintenance was associated with a significant RFS benefit compared with placebo (hazard ratio [HR], 0.515; 95% confidence interval [CI], 0.316–0.838; *p* = 0.0065). Approximately half of the enrolled patients (50.5%) had detectable MRD before or after transplantation, whereas patients without detectable MRD at either the pre-or post-transplant assessment did not show a clear benefit from gilteritinib maintenance (HR. 1.213; 95%CI, 0.616–2.387; *p* = 0.575). Subgroup tests were not adjusted for multiplicity. Grade ≥ 3 treatment-emergent adverse events occurred in 82.0% with gilteritinib vs. 53.1% with placebo, and 52.8% of gilteritinb recipients completed 24 months. Clinically relevant myelosuppression may occur, particularly when gilteritinib is administered concomitantly with azole antifungal agents. These findings support an MRD-guided approach to FLT3 inhibitor maintenance after allo-HCT and indicate that gilteritinib should not be considered broadly equivalent to sorafenib in the post-transplant setting. Rather, its potential role appears most convincing in patients with persistent or emerging molecular disease, with the optimal MRD threshold, timing and duration of intervention still requiring clarification [29]. This selected use is supported by current ELN-DAVID guidance for FLT3-ITD MRD detected by an ultrasensitive molecular assay [15].

Regarding quizartinib, the QuANTUM-First maintenance analysis is now available: no OS benefit was demonstrated among transplanted maintenance recipients, whereas benefit was observed among non-transplanted ones (HR, 0.401; 95%CI, 0.192–0.838). Patients were not re-randomized before maintenance, limiting the attribution of benefit to this phase alone [30].

In QuANTUM-R, patients assigned to quizartinib who underwent transplantation in CR and then received quizartinib maintenance appeared to have longer OS than those who did not receive maintenance [31]. However, these observations are based on very small post-transplant subsets and should be viewed as exploratory rather than practice-changing. Therefore, quizartinib remains a biologically plausible but insufficiently validated option for maintenance after transplant.

The addition of midostaurin to induction and consolidation chemotherapy has been shown to improve OS in patients with newly diagnosed FLT3-mutated AML [32]. However, data supporting its use as post-allo-HCT maintenance remain limited. In the small, open-label randomized RADIUS trial (*n* = 60; NCT01883362), designed to assess the safety and feasibility of midostaurin maintenance after transplantation, 18-month RFS was 89.0% with midostaurin vs. 76.0% with standard of care (HR, 0.46; 95%CI, 0.12–1.86; *p* = 0.27). Effective in vivo FLT3 inhibition was associated with improved RFS in an exploratory analysis. Moreover, treatment tolerability was suboptimal, with more than half of patients requiring dose modification or treatment discontinuation during the planned 1-year maintenance period: 16 of 30 assigned patients completed maintenance [29].

These limitations make midostaurin a less compelling maintenance candidate than sorafenib and highlight the importance of drug exposure, tolerability and pharmacodynamic inhibition when evaluating FLT3-directed strategies after allo-HCT (Table 1).

Overall, FLT3 inhibitor maintenance after allo-HCT represents the best-developed paradigm of targeted post-transplant therapy in AML, but the strength of evidence varies substantially across agents. Sorafenib currently has the most persuasive clinical support, whereas gilteritinib appears most relevant in an MRD-defined subgroup, and quizartinib and midostaurin remain less established. Treatment decisions should therefore be individualized according to the quality of supporting evidence, prior FLT3 inhibitor exposure, peri-transplant MRD status, tolerability, drug–drug interactions, GVHD risk and competing transplant-related complications. Prospective transplant-focused studies are still needed to define the optimal agent, timing, duration and biologically informed patient selection.

#### 3.1.2. IDH Inhibitors

In AML, IDH1 and IDH2 mutations represent recurrent molecular lesions and are also detected across other myeloid neoplasms, including myelodysplastic syndromes and chronic myelomonocytic leukemia [33].

Mutations in IDH1 or IDH2 confer a neomorphic enzymatic activity that leads to the accumulation of R-2-hydroxyglutarate, an oncometabolite that competitively inhibits α-ketoglutarate-dependent enzymes. This metabolic alteration promotes epigenetic dysregulation and disrupts normal hematopoietic differentiation [33,34,35,36].

The prognostic impact of IDH mutations in AML remains incompletely defined, with discordant results reported across studies [37].

Early studies have specifically evaluated outcomes after allo-HCT in patients with IDH-mutated AML. In a retrospective analysis of 99 patients with AML undergoing allo-HCT, 23 harbored IDH mutations: this subgroup had a 1-year relapse rate of 29% and, after adjustment for pre-transplant disease status, IDH1/2 mutation status was associated with an increased risk of post-transplant relapse (hazard ratio [HR], 2.8; *p* = 0.046) [38]. Chen et al. subsequently reported a larger multicenter retrospective analysis including 112 patients with IDH1- or IDH2-mutated AML undergoing transplantation. The 2-year cumulative incidences of relapse were 31.0% and 25.0% for IDH1/IDH2-mutated disease, respectively, with a 2-year progression-free survival of 58.0% among patients transplanted in CR1 or CR with incomplete recovery [39].

A subsequent analysis of 292 patients associated persistent IDH1 R132 or IDH2 R172, but not IDH2 R140, at transplant with relapse [40].

In the larger Pre-MEASURE analyses, isolated residual IDH1 in pre-transplant CR1 did not predict relapse or death (*n* = 148); residual IDH2 was associated with worse outcome (*n* = 257), particularly without baseline NPM1 or FLT3-ITD co-mutations [41].

Collectively, these findings indicate that IDH-mutated AML remains associated with clinically meaningful relapse risk after transplantation and provide a useful benchmark for maintenance studies, while also underscoring the need to clarify whether IDH mutation status has independent prognostic value in this setting.

The therapeutic landscape of IDH-mutated myeloid neoplasms has been substantially reshaped by the development of orally available targeted small-molecule inhibitors [42]. Ivosidenib—a selective IDH1 inhibitor—and enasidenib, a selective IDH2 inhibitor, reduce R-2-hydroxyglutarate levels and promote restoration of myeloid differentiation [43,44,45].

In large multicenter phase I/II studies, these agents showed favorable toxicity profiles and the ability to induce durable remissions, both as monotherapy and in combination regimens, in patients with relapsed/refractory and newly diagnosed AML. Based on these data, ivosidenib and enasidenib have been approved by the US Food and Drug Administration for IDH1- and IDH2-mutated AML, respectively [46,47].

Their US indications differ: both cover adult relapsed/refractory disease with the corresponding mutation; ivosidenib also covers selected newly diagnosed adults, alone or with azacitidine. According to current US prescribing information for ivosidenib and enasidenib, neither agent has an approved indication for post-transplant maintenance.

The biological rationale for IDH inhibitor maintenance after allo-HCT is therefore compelling, particularly because these agents are orally administered, mutation-directed, and generally well tolerated. However, clinical evidence in the post-transplant setting remains limited and largely investigational. Phase I/II trials have been evaluating ivosidenib (NCT03564821) and enasidenib for IDH2-mutated disease (NCT03728335 and NCT04522595). NCT03839771 evaluates a broader frontline treatment sequence rather than a dedicated post-allo-HCT maintenance strategy.

Fathi et al. reported the results of a phase I trial evaluating enasidenib as maintenance therapy after allo-HCT in patients with IDH2-mutated AML (NCT03515512). Two dose levels, 50 mg and 100 mg once daily, were assessed using a 3 + 3 dose-escalation design, with 10 additional patients treated at the recommended phase II dose (RP2D). Enasidenib was initiated between days 30 and 90 after transplantation and continued for twelve 28-day cycles. Twenty-three patients were enrolled, of whom 19 initiated post-HCT maintenance: 17 had AML and 2 had myelodysplastic syndrome (MDS). All but three patients were in CR1 at transplantation. No dose-limiting toxicities were observed, and the RP2D was established at 100 mg once daily. Treatment-attributable grade ≥ 3 toxicities were uncommon, with cytopenias being the most frequent. Eight patients discontinued maintenance before completing 12 cycles because of adverse events (*n* = 3), treatment for GVHD (*n* = 2), clinician decision (*n* = 1), relapse (*n* = 1), or COVID-19 infection (*n* = 1).

No cases of grade ≥ 3 acute GVHD were reported, and the 12-month cumulative incidence of moderate-to-severe chronic GVHD was 42% (95% CI, 20–63%). The cumulative incidence of relapse was 16% (95% CI, 3.7–36%), with only one relapse occurring during maintenance therapy. Two-year PFS and OS were 69% (95% CI, 39–86%) and 74% (95% CI, 44–90%), respectively. These findings suggest that enasidenib maintenance after allo-HCT is feasible and generally well tolerated, with preliminary evidence of antileukemic activity. Nevertheless, the limited sample size and non-comparative design preclude definitive conclusions regarding efficacy and require validation in larger prospective studies [45].

A subsequent two-center pilot study treated 15 patients with enasidenib 100 mg daily from days 50–120 after transplant for up to 2 years (NCT03728335). Twelve completed all 24 cycles; 2-year OS and leukemia-free survival were both 100%, and chronic GVHD-free–relapse-free survival was 80.0%. These estimates concern selected patients in remission with hematologic recovery and without grade ≥ 2 acute GVHD and require confirmation in controlled studies [48].

Fathi et al. also conducted a multicenter phase I trial evaluating ivosidenib maintenance after allo-HCT in patients with IDH1-mutated AML (NCT03564821). Ivosidenib was initiated between days 30 and 90 after transplantation and administered for up to twelve 28-day cycles. The initial dose level was 500 mg once daily, with planned de-escalation to 250 mg once daily if required, according to a 3 + 3 dose de-escalation design. Ten additional patients were planned to receive the maximum tolerated dose (MTD) or RP2D. The primary endpoint was to establish the MTD or RP2D of post-transplant ivosidenib. Eighteen patients were enrolled and 16 initiated maintenance therapy. One dose-limiting toxicity, grade 3 QTc prolongation, was observed; consequently, the RP2D was established at 500 mg once daily. Treatment-attributable grade ≥ 3 adverse events were infrequent, with QTc prolongation being the most common severe event, reported in two patients. Eight patients discontinued maintenance, only one because of an adverse event. The 6-month cumulative incidence of grade 2–4 acute GVHD was 6.3%, whereas the 2-year cumulative incidence of chronic GVHD was 63%. The 2-year cumulative incidences of relapse and non-relapse mortality were 19% and 0%, respectively. Two-year PFS and OS were 81% and 88%, respectively [49].

The authors concluded that ivosidenib was safe and well tolerated as maintenance therapy after allo-HCT. The low cumulative incidence of relapse and absence of non-relapse mortality, together with encouraging estimates of PFS and OS, support further evaluation of this strategy. Nevertheless, as observed for enasidenib, these results should be interpreted as preliminary because they derive from an early-phase, non-randomized study with limited patient numbers.

Taken together, the available data suggest that IDH inhibitor maintenance after allo-HCT is feasible and generally tolerable, but the evidence remains insufficient to define this approach as a standard of care. Existing studies were primarily designed to assess safety and feasibility rather than comparative efficacy, and their interpretation is limited by small sample sizes, non-randomized design, and heterogeneous patient populations. Several clinically relevant questions remain unresolved, including the optimal duration of treatment, the effect of maintenance on molecular relapse, the prognostic significance of persistent or cleared IDH mutations at recurrence, and the interaction between differentiation-based antileukemic activity and post-transplant immune dynamics. Thus, IDH inhibitors remain a rational but narrowly applicable maintenance strategy, dependent on the presence of a defined molecular lesion and still awaiting prospective validation. This limitation naturally shifts attention to broader maintenance approaches that are not restricted to a single targetable mutation (Table 2).

### 3.2. Epigenetic Modifiers

#### Hypomethylating Agents as Single-Agent Maintenance

Hypomethylating agents (HMAs) are an established therapeutic class in myeloid malignancies and have been extensively investigated as maintenance therapy after allo-HCT because of their broad antileukemic and immunomodulatory properties [50].

Unlike mutation-directed agents, HMAs are not restricted to a defined molecular subgroup. This broad applicability is attractive in the post-transplant setting, but it also complicates patient selection and makes clinical benefit difficult to attribute to a specific biological mechanism.

The rationale for HMAs maintenance after allo-HCT relies on epigenetic reprogramming, direct antileukemic activity, and the potential enhancement of graft-versus-leukemia responses. These effects may include DNA hypomethylation, re-expression of silenced tumor suppressor genes, modulation of antigen presentation, and changes in T-cell and natural killer cell compartments. However, the extent to which these mechanisms translate into durable relapse reduction remains uncertain, particularly because HMA exposure after transplantation is often limited by cytopenias, infections, gastrointestinal toxicity, and treatment discontinuation [51,52,53].

Early clinical studies mainly established feasibility and dosing rather than definitive efficacy. In a phase I trial, de Lima et al. evaluated low-dose azacitidine after allo-HCT in 45 high-risk patients with AML or MDS using a Bayesian adaptive design. Azacitidine was tested at 8–40 mg/m^2^ daily for 5 days, with schedules ranging from one to four cycles. Reversible thrombocytopenia was dose-limiting, and 32 mg/m^2^ daily for 5 days over four cycles were identified as the preferred regimen. One-year EFS and OS were 58% and 77%, respectively, but no clear dose-dependent effect on global DNA methylation was observed. Thus, the study supported the feasibility of low-dose azacitidine maintenance, while providing no conclusive evidence of efficacy [50].

Goodyear et al. further supported the tolerability of post-transplant azacitidine and provided mechanistic evidence of immune modulation. At 36 mg/m^2^ daily, azacitidine expanded regulatory T cells and induced cytotoxic CD8+ T-cell responses against tumor-associated antigens, suggesting that HMAs may influence graft-versus-leukemia activity beyond direct cytotoxic or epigenetic effects [52].

Decitabine has also been evaluated as post-allo-HCT maintenance. In a phase I dose-escalation study, Pusic et al. treated 24 patients with AML or MDS in first complete remission with decitabine 5–15 mg/m^2^/day for 5 consecutive days every 6 weeks, for up to eight cycles. The maximum tolerated dose was not reached, but grade 3–4 hematologic toxicity occurred in 75% of patients, mainly at the highest dose. Nine patients completed all planned cycles, and eight remained in complete remission. Although low-dose decitabine was feasible and did not appear to exacerbate GVHD, the study was small and non-comparative, precluding firm conclusions regarding relapse prevention [54].

A biologically distinct strategy combines decitabine with granulocyte colony-stimulating factor (G-CSF), based on the hypothesis that G-CSF may drive leukemic cells into cycle and thereby increase their susceptibility to the cell cycle–dependent activity of decitabine [55].

In a multicenter, open-label, randomized phase II trial, Gao et al. [56]. compared recombinant human G-CSF plus low-dose decitabine (5 mg/m^2^; G-Dec) with observation in 204 MRD-negative patients with AML after transplantation. G-Dec significantly reduced the 2-year cumulative incidence of relapse compared with observation (15.0% vs. 38.3%; HR, 0.32; 95% CI, 0.18–0.57; *p* < 0.01), without an apparent increase in chronic GVHD (23.0% vs. 21.7%; HR, 1.07; 95% CI, 0.60–1.92; *p* = 0.81). The expansion of NK cells, CD8+ T cells, and regulatory T cells supported a possible immunomodulatory contribution. Nevertheless, these findings remain correlative, and the relative contribution of pharmacologic priming, direct antileukemic activity, and immune modulation cannot be disentangled. Moreover, the open-label phase II design and enrollment of MRD-negative patients limit generalizability [56].

Thus, rhG-CSF plus low-dose decitabine is a promising but still investigational maintenance strategy after allo-HCT. Larger studies are needed to define optimal patient selection; treatment duration; and the balance between relapse prevention, tolerability, and immune-mediated effects.

The largest randomized trial of azacitidine maintenance after allo-HCT enrolled 187 patients with high-risk AML or MDS in complete remission and compared azacitidine with observation [57]. Azacitidine was planned at 32 mg/m^2^/day subcutaneously for 5 days every 28 days for up to 12 cycles. However, treatment exposure was limited: although 87 of 93 patients assigned to azacitidine started therapy, the median number of administered cycles was only four. In the published efficacy analysis, which excluded six randomized patients who never started azacitidine, azacitidine did not significantly improve RFS (median RFS, 2.07 vs. 1.28 years; *p* = 0.43) or OS (median OS, 2.52 vs. 2.56 years; *p* = 0.85), and multivariable analyses did not show a significant benefit. RFS and OS were measured from transplant: 24 of 87 treated patients completed all 12 cycles, and the study closed below its planned accrual [57].

The study therefore demonstrated feasibility and general safety but failed to establish efficacy and highlighted treatment deliverability as a central limitation of HMA-based maintenance.

Overall, single-agent HMA maintenance after allo-HCT remains biologically plausible and clinically feasible in selected patients, but its efficacy is not firmly established. The available evidence is limited by small sample sizes, heterogeneous populations, variable schedules, inconsistent MRD integration, and incomplete treatment exposure. These limitations make it difficult to define which patients are most likely to benefit and whether negative results reflect insufficient biological activity, inadequate drug delivery, or suboptimal patient selection.

A systematic review and meta-analysis by Kungwankiattichai et al. evaluated HMA maintenance after allo-HCT in patients with AML and provides a broader, though heterogeneous, summary of the available evidence [58].

Fourteen studies, including two randomized trials, met the eligibility criteria. Compared with observation, HMA maintenance was associated with superior OS and RFS, with pooled risk ratios of 1.38 and 1.46, respectively, and with a lower cumulative incidence of relapse. Non-relapse mortality was also lower in the HMA group, while the incidences of grade 3–4 acute GVHD and chronic GVHD were not significantly different between groups. In subgroup analyses, decitabine maintenance appeared to be associated with a lower rate of chronic GVHD than observation alone. However, these findings should be interpreted cautiously because the included studies were heterogeneous and largely non-randomized, with variable patient selection, dosing schedules, and treatment duration. The pooled risk ratios refer to survival proportions, not hazards; the lower non-relapse mortality remains an association susceptible to selection and confounding [58].

Oral HMA formulations have been developed to improve feasibility by reducing the logistical burden of repeated parenteral administration and potentially prolonging drug exposure. However, oral administration does not necessarily overcome the major barriers to post-transplant maintenance, which include cytopenias, infectious complications, treatment interruptions, and uncertain antileukemic efficacy.

Oral azacitidine (CC-486) was designed to extend HMA exposure and has been evaluated as post-allo-HCT maintenance. In a phase I/II study, de Lima et al. treated 30 adults with AML or MDS in morphologic CR, starting CC-486 42–84 days after transplantation using 7- or 14-day schedules within 28-day cycles for up to 12 cycles. Grade 3–4 treatment-emergent adverse events occurred in 22/30 (73.0%) patients, predominantly gastrointestinal or hematologic; these were not all attributed to CC-486. Thirteen patients completed all 12 cycles, and transplant-related mortality was 3.0% [59].

Among 28 evaluable patients, relapse or progression occurred in 21%, with fewer events in the 14-day than in the 7-day cohort (13% vs. 43%). Although these data support feasibility and suggest a possible relapse-control signal, interpretation is constrained by the small non-randomized cohort, short follow-up, and potential schedule-related selection effects. CC-486 therefore remains investigational, and randomized validation is required to determine whether prolonged oral HMA exposure improves post-transplant outcomes [59]. The survival benefit of CC-486 in QUAZAR AML-001 was demonstrated in patients not proceeding to transplantation and does not establish post-allo-HCT efficacy [60].

The phase III randomized, placebo-controlled AMADEUS trial evaluated CC-486 as maintenance therapy after allo-HCT (NCT04173533). Follow-up was completed in April 2026 (326 participants): no registry results were posted as of Semptember 2026, and no outcome publication had been identified by that date.

Decitabine/cedazuridine is an orally available HMA formulation initially approved by the Food and Drug Administration for myelodysplastic syndromes, including CMML, on pharmacokinetic bridging evidence [61].

In May 2026, a separate US indication was added with venetoclax for newly diagnosed AML in adults aged ≥75 years or with comorbidities precluding intensive induction; this does not establish post-transplant maintenance.

Its potential role after allo-HCT is supported by both practical and biological considerations: oral administration may improve feasibility, while decitabine may contribute to relapse prevention through epigenetic antileukemic activity, enhancement of graft-versus-leukemia effects, and natural killer cell-mediated antibody-dependent cellular cytotoxicity against AML blasts [62].

Nevertheless, clinical evidence in the post-transplant setting remains limited, and reported off-protocol experience in high-risk MDS should be interpreted cautiously.

In an 18-patient retrospective MDS cohort, 2-year RFS and OS were 66.7% and 72.2% respectively [63].

The single-arm GFM-DACORAL-DLI study-initiated maintenance in 34/51 transplanted patients; 14 completed the cycles, and seven also received donor lymphocyte infusion (DLI). Among the first 28 treated patients, 1-year DFS from transplant was 70.4% (95% CI, 55.1–89.9); grade ≥ 3 hematologic events occurred in 74.0%, with one treatment-related death. The concomitant DLI and non-randomization design limit attribution of outcomes to the drug alone [64].

Further prospective studies, including NCT04980404, are expected to clarify the safety, feasibility, and potential efficacy of oral decitabine/cedazuridine as maintenance after allo-HCT.

Guadecitabine is a next-generation hypomethylating agent consisting of decitabine linked to deoxyguanosine, a structural modification that protects the molecule from cytidine deaminase-mediated degradation and permits gradual decitabine release after subcutaneous administration. This pharmacologic profile may prolong drug exposure and enhance epigenetic activity. Despite this rationale, guadecitabine did not improve overall survival compared with available treatment options in the ASTRAL studies, and the post-transplant investigators subsequently reported discontinuation drug supply [65,66,67].

Oran et al. reported a single-center phase II trial evaluating guadecitabine—with or without donor lymphocyte infusion—as maintenance, pre-emptive, or salvage therapy for AML and MDS after transplantation. Patients were allocated to three cohorts according to post-transplant disease status: hematologic relapse (cohort 1, *n* = 13), MRD positivity (cohort 2, *n* = 18), or remission without MRD within 100 days after transplantation (cohort 3, *n* = 24). The primary endpoints were morphological complete remission in cohort 1 and MRD eradication in cohort 2 within six cycles; patients in cohort 3 received up to 12 cycles with the aim of improving relapse-free survival. Morphological complete remission was achieved in 3/13 patients in cohort 1, whereas 8/17 (47.1%) patients in cohort 2 achieved MRD eradication; among patients who cleared MRD, the 2-year relapse-free survival rate was 62.5%. In cohort 3, the 2-year relapse-free survival rate was likewise 62.5% after a median follow-up of 48 months [68].

No unexpected adverse events or graft failures were reported. Overall, guadecitabine demonstrated a favorable safety profile and preliminary activity across post-transplant settings; however, its role remains investigational and is further constrained by discontinuation of the drug (Table 3).

### 3.3. Hypomethylating Agents in Combination

Combination strategies aim to enhance the antileukemic and immunomodulatory effects of HMAs, but their evaluation after allo-HCT is particularly challenging. In this setting, incremental efficacy must be weighed against overlapping myelosuppression, infectious risk, treatment discontinuation, drug interactions, and possible effects on graft-versus-leukemia activity. Therefore, biological plausibility alone is insufficient; combinations require prospective evidence of tolerability, sustained exposure, and net clinical benefit.

Among explored approaches, azacitidine has been combined with gemtuzumab ozogamicin, an anti-CD33 antibody–drug conjugate, to increase antileukemic activity with reported 1-year DFS and OS rates around 60% to 70%, respectively [68,69].

Retrospectively matched controls had 1-year DFS and OS of 42.8% and 59.8%; differences were not significant (*p* = 0.222 and *p* = 0.138, respectively). However, the absence of a randomized control arm, short treatment duration, and limited sample size preclude the determination of whether these outcomes reflect additive drug activity, patient selection, or graft-versus-leukemia effects. Other combinations, including HMAs with venetoclax or APR-246, are also under investigation but remain experimental [68].

For venetoclax plus azacitidine, a phase I study initiated maintenance in 22/27 transplanted patients, with 2-year OS and PFS of 67% and 59%. All received venetoclax-containing conditioning [70]. A subsequent cohort using post-transplant cyclophosphamide initiated maintenance in 17/23 patients; six completed all eight cycles, and 15/17 required venetoclax dose reduction [71]. These non-randomized studies support feasibility in selected patients but cannot isolate the maintenance effect.

The randomized phase III VIALE-T trial (NCT04161885) compared venetoclax plus azacitidine for up to six cycles, followed by venetoclax for up to 24 cycles in total, with the best supportive care. Among 430 randomized patients, registry results reported an OS HR of 1.20 (95% CI, 0.81–1.77). The study was terminated for strategic considerations before the required event count was reached, and no formal hypothesis test was performed. The results do not demonstrate benefit, but also do not establish excess mortality (ClinicalTrials.gov, 2026).

For TP53-mutated AML or MDS, a single-arm phase II study treated 33 patients with eprenetapopt (APR-246) plus azacitidine after allo-HCT; 1-year RFS and OS were 59.9% and 78.8%. These preliminary estimates require confirmation with a concurrent comparator [72].

Combinations of HMAs with FLT3 or IDH inhibitors have not yet been specifically validated as post-allo-HCT maintenance, despite their established use in frontline or disease-directed settings. Extrapolation to the post-transplant context is therefore premature. As an alternative to universal maintenance, MRD-guided pre-emptive treatment may offer a more selective strategy. In the phase II REALAZA2 trial, azacitidine initiated at MRD detection in patients with AML or MDS was associated with delayed overt relapse, with 58% of patients remaining relapse-free and alive 6 months after treatment initiation in the initial report [73].

The final long-term report expanded the treated cohort from 53 to 95 patients after intensive chemotherapy alone or subsequent allo-HCT: 60/95 (63.0%; 95% CI, 54–71) were alive and relapse-free at 6 months. Among 60 early MRD responders, 31 maintained responses without hematologic relapse for at least 2 years [74].

These findings support the concept of MRD-triggered intervention, but they do not establish superiority over fixed-duration maintenance. Its efficacy will depend on assay sensitivity, monitoring frequency, timing of treatment initiation, disease kinetics, and prospective validation in controlled studies. Figure 3 summarized the main medications used for post-transplant maintenance.

#### Menin Inhibitors

Menin inhibitors represent an emerging molecularly targeted strategy for post-transplant maintenance in AML harboring *KMT2A* rearrangements or *NPM1* mutations. These leukemias depend on the interaction between menin and KMT2A to maintain aberrant transcriptional programs, providing a strong biological rationale for suppressing residual disease after allo-HCT [75,76]. Revumenib and ziftomenib have demonstrated antileukemic activity in relapsed or refractory AML, but evidence in the maintenance setting remains preliminary [75,76,77,78,79]. Revumenib is currently being evaluated after allo-HCT in the phase I trial NCT06575296, while ziftomenib is under investigation in the prospective phase I study NCT06440135. Importantly, in the post-transplant setting, safety and tolerability represent critical determinants of clinical applicability rather than secondary endpoints. Potential myelosuppression, infections, QTc prolongation, differentiation syndrome, hepatic toxicity and clinically relevant drug–drug interactions may overlap with early transplant-related complications, concomitant immunosuppression and anti-infective prophylaxis. Even moderate toxicity may impair hematopoietic and immune recovery, increase treatment interruptions or dose reductions, exacerbate competing transplant-related morbidity and limit the sustained exposure required for effective maintenance. Consequently, post-transplant studies should carefully define the optimal starting time, maintenance-specific dose, and duration, rather than extrapolating schedules developed for overt relapsed or refractory disease. They should also evaluate possible effects on GVHD, graft-versus-leukemia activity and immune reconstitution. Therefore, despite a compelling molecular rationale, menin inhibitors should currently be regarded as an investigational, biomarker-selected maintenance approach whose clinical value will depend not only on relapse prevention but also on a favorable and demonstrable post-transplant therapeutic index.

## 4. TP53-Mutated Acute Myeloid Leukemia

*TP53*-mutated AML represents one of the most challenging settings for post-transplant relapse prevention; this is because allo-HCT may provide the only potentially curative option but is frequently followed by early recurrence, particularly in patients with persistent molecular disease, complex or monosomal karyotype, or inadequate pre-transplant disease control [80]. This adverse biology provides a strong rationale for maintenance as prophylaxis more than MRD-guided pre-emptive therapy, although no strategy has yet been established as the standard of care. Hypomethylating agents have been most extensively explored because of their relative tolerability and potential immunomodulatory effects, but randomized data have not demonstrated a consistent survival benefit with unselected post-transplant azacitidine, and any specific advantage in *TP53*-mutated AML remains uncertain. Eprenetapopt, a p53-reactivating agent, combined with azacitidine, was evaluated in a single-arm phase II study of 33 patients with *TP53*-mutated AML or MDS after allo-HCT; the reported 1-year relapse-free and overall survival rates were 59.9% and 78.8%, respectively. Although encouraging, these outcomes require cautious interpretation because of the absence of a concurrent comparator and a limited cohort size and potential selection of patients able to initiate maintenance. Furthermore, subsequent development of eprenetapopt has been tempered by negative results outside the post-transplant setting, limiting extrapolation of this preliminary signal. Other approaches—including decitabine-based regimens, venetoclax combinations, immune-modulating strategies and agents directed against adverse-risk or leukemic stem cell compartments—remain investigational and may be constrained by cytopenias, infections and impaired immune recovery. Consequently, post-allo-HCT therapy in *TP53*-mutated AML should currently be considered an individualized and preferably trial-based strategy, integrating molecular MRD kinetics, cytogenetic complexity, remission status, hematopoietic recovery, GVHD, and competing transplant-related toxicity. Future studies should use molecularly defined cohorts and concurrent controls to determine whether maintenance can eradicate residual *TP53*-mutated clones rather than merely delay overt relapse.

## 5. Conditioning Intensity and the Rationale for Post-Transplant Intervention

Conventional myeloablative conditioning (MAC) is generally associated with lower relapse rates than reduced-intensity conditioning (RIC) but is usually indicated for younger and medically fit patients, due to its regimen-related toxicity that limits its applicability in older patients or in those with clinically relevant comorbidities [81].

Conversely, reduced-intensity approaches may be less effective in patients with persistent disease at transplantation. As reported by Hourigan et al., the use of RIC rather than MAC in patients with active disease at the time of allo-HCT, even in those with only measurable residual disease positivity alone, is associated with an increased risk of post-transplant relapse [82].

These findings highlight the rationale for post-transplant intervention in patients receiving RIC, in whom lower regimen-related toxicity may be offset by a higher relapse risk. The key therapeutic goal is therefore to preserve the tolerability of attenuated conditioning while improving disease control through rationally integrated post-transplant maintenance strategies.

Within this framework, the pharmacokinetic targeting of busulfan exposure combined with post-transplant azacitidine has been investigated as a strategy for reducing relapse risk without increasing non-relapse mortality. In this study, the primary endpoint was 2-year progression-free survival. Eligible patients included individuals aged 60–74 years with AML in first complete remission, patients younger than 75 years with MDS, candidates with an available matched sibling or volunteer unrelated donor, and patients without significant pre-transplant end-organ dysfunction. The study completed accrual in October 2013 and enrolled 63 patients [83].

The feasibility limitations of this approach are further illustrated by the phase II National Cancer Institute/Alliance trial (Cancer and Leukemia Group B 100801), which enrolled 66 patients; of these, only 42 initiated azacitidine maintenance and only 17 completed all six planned cycles [84].

These findings reinforce a consistent theme across HMA-based maintenance studies: although feasibility can be demonstrated in selected patients, treatment deliverability, cumulative toxicity and incomplete exposure remain major barriers to establishing broad clinical efficacy after allo-HCT.

## 6. GVHD Prophylaxis and Treatment and Maintenance Therapy Post-allo-HCT

GVHD prophylaxis represents an additional determinant of post-transplant relapse risk because strategies that effectively prevent alloreactivity may also attenuate graft-versus-leukemia activity [85]. Calcineurin inhibitor-based regimens combined with methotrexate or mycophenolate mofetil remain widely used, but their prolonged immunosuppressive effect may delay immune reconstitution and potentially limit antileukemic surveillance. In vivo T-cell depletion with antithymocyte globulin or alemtuzumab reduces GVHD, particularly after unrelated-donor transplantation, but deeper and more sustained T-cell suppression may increase infectious complications and weaken graft-versus-leukemia effects, especially when higher doses or prolonged exposure are used [86]. By contrast, post-transplant cyclophosphamide selectively targets proliferating alloreactive T cells while preserving broader immune recovery and has enabled the expansion of haploidentical and mismatched transplantation. However, whether it preserves graft-versus-leukemia activity more effectively than conventional prophylaxis remains uncertain, as relapse outcomes are influenced by donor type, conditioning intensity, graft source, disease status and measurable residual disease. More intensive approaches, including combinations of post-transplant cyclophosphamide with tacrolimus and mycophenolate mofetil, may further reduce acute and chronic GVHD but could also delay immune reconstitution and narrow the therapeutic window for early maintenance. Therefore, GVHD prevention and relapse control should not be viewed as independent objectives: the optimal regimen should minimize clinically relevant GVHD without abolishing beneficial donor alloreactivity [86,87,88,89]. Future maintenance studies should stratify patients according to GVHD prophylaxis and prospectively incorporate immune-reconstitution, MRD, GVHD and relapse endpoints to determine whether the efficacy and tolerability of post-transplant interventions vary across immunosuppressive platforms.

When GVHD develops during maintenance therapy, management should be individualized according to GVHD severity, the suspected contribution of the maintenance agent, hematologic recovery, infectious risk and relapse risk. Maintenance may generally be continued with close monitoring in mild, controlled GVHD when organ function and blood counts remain adequate; however, treatment should be interrupted in clinically significant acute or chronic GVHD requiring intensified systemic immunosuppression, particularly in the presence of cytopenias, infection or organ toxicity. Reintroduction at the same or a reduced dose may be considered after GVHD stabilization and recovery from treatment-related complications. Decisions should be multidisciplinary and should account for drug–drug interactions with immunosuppressive and anti-infective agents because evidence supporting a uniform approach remains limited.

Finally, graft source may influence the balance between graft-versus-leukemia activity and transplant-related toxicity, particularly in patients at high risk of relapse. Peripheral blood stem cell grafts contain higher numbers of donor immune cells and may provide faster hematopoietic recovery and stronger antileukemic activity than bone marrow grafts, but at the cost of a greater risk of chronic GVHD. Bone marrow may reduce chronic GVHD burden, although its potentially lower immunologic intensity should be considered in patients with adverse disease biology or persistent MRD. Umbilical cord blood offers rapid availability and permits greater HLA disparity, with potentially favorable antileukemic effects in selected high-risk settings, but delayed engraftment, infectious complications and higher early non-relapse mortality may limit its applicability. However, comparisons among graft sources remain strongly confounded by donor availability, HLA matching, conditioning intensity, GVHD prophylaxis, disease status and center experience. Graft source should therefore be regarded as an integral component of relapse-risk management rather than an isolated technical choice. Prospective studies should clarify whether selected high-risk or MRD-positive patients benefit from graft sources capable of enhancing graft-versus-leukemia activity while maintaining an acceptable balance between disease control, GVHD, immune recovery and transplant-related toxicity.

## 7. Future Directions

Future development of post-transplant maintenance in AML is increasingly moving beyond single-agent empiric approaches toward molecularly selected, mechanism-based and biomarker-adapted strategies. This expanding therapeutic pipeline includes epigenetic agents, menin inhibitors, BCL-2–directed combinations, IDH-directed therapies and novel agents with putative activity against adverse-risk or stem cell-enriched leukemic compartments. However, the field remains characterized by a substantial gap between biological plausibility and clinical validation. Most ongoing studies are early-phase; single-arm; or primarily designed to establish feasibility, dose and safety rather than comparative efficacy. Accordingly, future evidence should be interpreted within the specific challenges of the post-transplant setting, where any relapse-preventive intervention must achieve sustained antileukemic activity without impairing immune reconstitution, exacerbating graft-versus-host disease, increasing infectious risk, or limiting deliverability through cumulative toxicity.

Among emerging epigenetic approaches, histone deacetylase inhibition has generated interest as a potential strategy to modulate residual leukemic survival and signaling after transplantation. Chidamide, in particular, has shown preclinical activity against AML cells, with proposed mechanisms including SOCS3 upregulation and the attenuation of JAK2/STAT3 signaling.

These observations provide a biologically coherent rationale for post-allo-HCT maintenance; however, the translational relevance of this mechanism remains uncertain. In the post-transplant milieu, drug activity may be modified by immune reconstitution, concurrent immunosuppression, cytopenias, infections and competing transplant-related toxicities. Therefore, HDAC inhibition should currently be regarded as an investigational approach requiring prospective validation rather than as an established extension of epigenetic maintenance.

Chidamide maintenance is being evaluated in the multicenter phase II trial NCT07304232 in patients with intermediate- or high-risk AML who are in complete remission at transplantation. The drug is administered orally at 10 mg once daily on days 1–5 of each week in 28-day cycles, with treatment planned for up to 24 months and dose interruption or modification allowed for toxicity. The prolonged planned exposure reflects the hypothesis that sustained epigenetic modulation may be required to influence relapse biology. At the same time, it raises important questions regarding treatment adherence, cumulative toxicity, the management of low-level or molecular recurrence during therapy, and the interpretation of relapse events occurring under ongoing maintenance.

The combination of chidamide with azacitidine, under investigation in NCT05270200, represents a rational attempt to intensify epigenetic pressure in high-risk AML after allo-HCT. Nevertheless, combination maintenance should not be assumed to improve outcome solely based on complementary mechanisms. In this context, overlapping myelosuppression, infectious complications, treatment discontinuation and potential effects on graft-versus-leukemia activity are central determinants of net clinical benefit. Prospective studies will therefore need to demonstrate not only biological activity, but also sustained treatment exposure and clinically meaningful relapse reduction.

Menin inhibition represents a particularly relevant direction for molecularly defined AML subsets, especially those driven by KMT2A rearrangements or NPM1 mutations. Post-transplant maintenance with revumenib is being evaluated in the phase I trial NCT06575296, which aims to define safety, tolerability, dose and preliminary efficacy in this setting. The biological rationale is strong, given the dependence of these leukemias on menin-mediated transcriptional programs. However, post-transplant use raises specific questions that differ from those encountered in relapsed or refractory disease, including whether menin inhibition can eradicate residual leukemic clones rather than delay recurrence, how it should be integrated with MRD surveillance, and whether activity observed in overt relapse is transferable to maintenance during remission.

Ziftomenib is being explored with a similar rationale in NCT06440135, a prospective multicenter open-label phase I study that includes dose escalation followed by an expansion cohort. Its potential role lies in targeting residual menin-dependent transcriptional programs, including leukemic stem cell-associated states, while potentially complementing graft-versus-leukemia activity. Nonetheless, the study is primarily designed to establish feasibility, dose and safety rather than definitive relapse-preventive efficacy. Early antileukemic signals will therefore require cautious interpretation, particularly because of molecular patient selection, the absence of randomized comparison and the difficulty of separating pharmacologic effects from post-transplant immune dynamics.

Strategies based on apoptotic priming constitute another area of clinical development. Lisaftoclax, an investigational BCL-2 inhibitor, has shown preclinical synergy with hypomethylating agents in myelodysplastic syndromes, providing the basis for evaluation with azacitidine as post-allo-HCT maintenance in high-risk AML. The multicenter, open-label, randomized trial NCT07686965 is therefore noteworthy because randomized designs are still uncommon in this field. However, extrapolation from preclinical MDS models to AML maintenance after transplantation remains uncertain. Moreover, BCL-2 inhibition in the early post-transplant period may be limited by cytopenias, infections and treatment interruptions, making safety, deliverability and sustained exposure key endpoints alongside relapse prevention.

Novel agents with activity against biologically adverse or stem cell–enriched leukemic compartments may also broaden the maintenance landscape. ONC201, a first-in-class imipridone, acts as a selective antagonist of the G protein-coupled receptor DRD2 and as an allosteric agonist of mitochondrial caseinolytic protease P. Preclinical studies have suggested activity against leukemia cells—including models with TP53 mutation and complex karyotype—and preferential effects on leukemia stem cells while sparing normal marrow cells. These features make ONC201 conceptually attractive after allo-HCT, particularly for patients lacking established targetable lesions. Nevertheless, its broad mechanism of action also complicates interpretation, and it remains to be determined whether preclinical stem cell activity can translate into clinically meaningful suppression of residual disease in the post-transplant setting.

In a phase I 3 + 3 dose-escalation trial (NCT03932643), ONC201 was evaluated as post-transplant maintenance in AML, with dose-limiting toxicities during cycle 1 and grade ≥ 3 adverse events as key safety objectives. Twenty patients were enrolled, with a median age of 68 years. ONC201 was administered orally once weekly for up to thirteen 4-week cycles, with dose escalation from 250 mg to 625 mg weekly. No dose-limiting toxicities or graft failures were observed, and the reported safety profile supported the feasibility of weekly oral administration. However, the cohort was small and heterogeneous, the study lacked a comparator arm, and efficacy was not a primary objective. ONC201 should therefore be considered an investigational platform rather than an established maintenance strategy. Future development will likely require biomarker-defined patient selection and carefully designed combination studies, particularly with agents such as azacitidine, to determine whether added biological activity translates into relapse reduction without excessive toxicity.

Olutasidenib, an orally available inhibitor of mutant IDH1, is also being explored as a post-transplant maintenance strategy for patients with IDH1-mutated AML. By inhibiting mutant IDH1 activity, olutasidenib may reduce R-2-hydroxyglutarate-driven leukemogenic signaling, promote differentiation, and suppress residual leukemic clones after allo-HCT. The phase I trial NCT06543381 is evaluating its safety, tolerability, toxicity profile and preliminary efficacy in this context. Although the rationale is consistent with other IDH-directed strategies, the clinical role of olutasidenib remains investigational and will require prospective evidence demonstrating durable relapse prevention without compromising post-transplant tolerability or immune recovery (Table 4).

## 8. Conclusions

Overall, the next phase of post-transplant maintenance research in AML should prioritize prospective, adequately powered, and biologically stratified studies that move beyond feasibility toward clinically meaningful endpoints. Future trials should integrate standardized MRD monitoring, molecular risk assessment, transplant-related variables and immune-reconstitution parameters to define not only whether maintenance reduces relapse, but also in whom, when, and for how long it should be administered. Equally important will be the distinction between fixed-duration maintenance and MRD-triggered pre-emptive intervention, as these approaches may differ substantially in therapeutic intent, toxicity burden and resource utilization.

A further unresolved issue concerns the appropriate dose intensity of maintenance treatment. Because post-allo-HCT maintenance is intended primarily as relapse prophylaxis during remission, rather than as treatment of overt active disease, it should not be automatically extrapolated from the full therapeutic doses used in relapsed/refractory or frontline disease-directed settings. The optimal maintenance dose should instead be defined by the minimum exposure capable of sustaining antileukemic or immunomodulatory activity while preserving hematopoietic recovery, limiting cumulative toxicity, avoiding treatment discontinuation and maintaining the delicate balance between graft-versus-leukemia effects and graft-versus-host disease. Ultimately, post-allo-HCT maintenance should evolve from a uniform relapse-prevention strategy into a personalized, dynamically adapted intervention capable of complementing graft-versus-leukemia activity while preserving tolerability and transplant-related immune recovery.

## Figures and Tables

**Figure 1:**
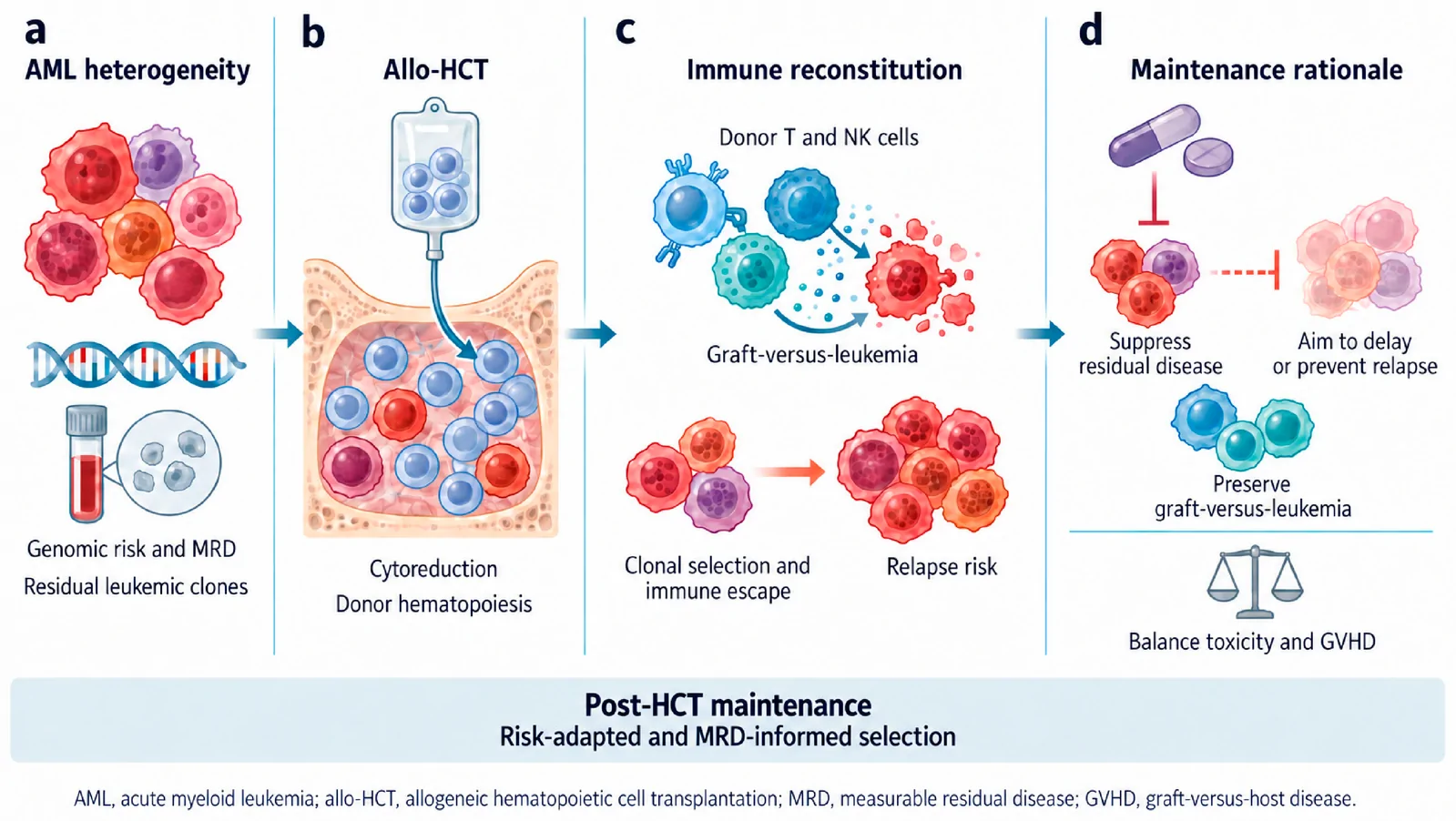
Conceptual framework for post-transplant maintenance in AML. Advances in genomic classification, MRD assessment and targeted therapies have improved AML risk stratification and treatment selection, but durable disease control remains limited by clonal heterogeneity, adaptive resistance and residual leukemic burden (**a**). Allo-HCT provides a potentially curative platform through cytoreduction (**b**) and graft-versus-leukemia activity (**c**), yet relapse remains the major cause of treatment failure. Post-transplant maintenance strategies aim to suppress residual disease during immune reconstitution, delay or prevent relapse, and preserve graft-versus-leukemia effects while minimizing toxicity and GVHD (**d**). This framework supports a risk-adapted and MRD-informed approach rather than uniform use of maintenance therapy in all transplanted patients.

**Figure 2:**
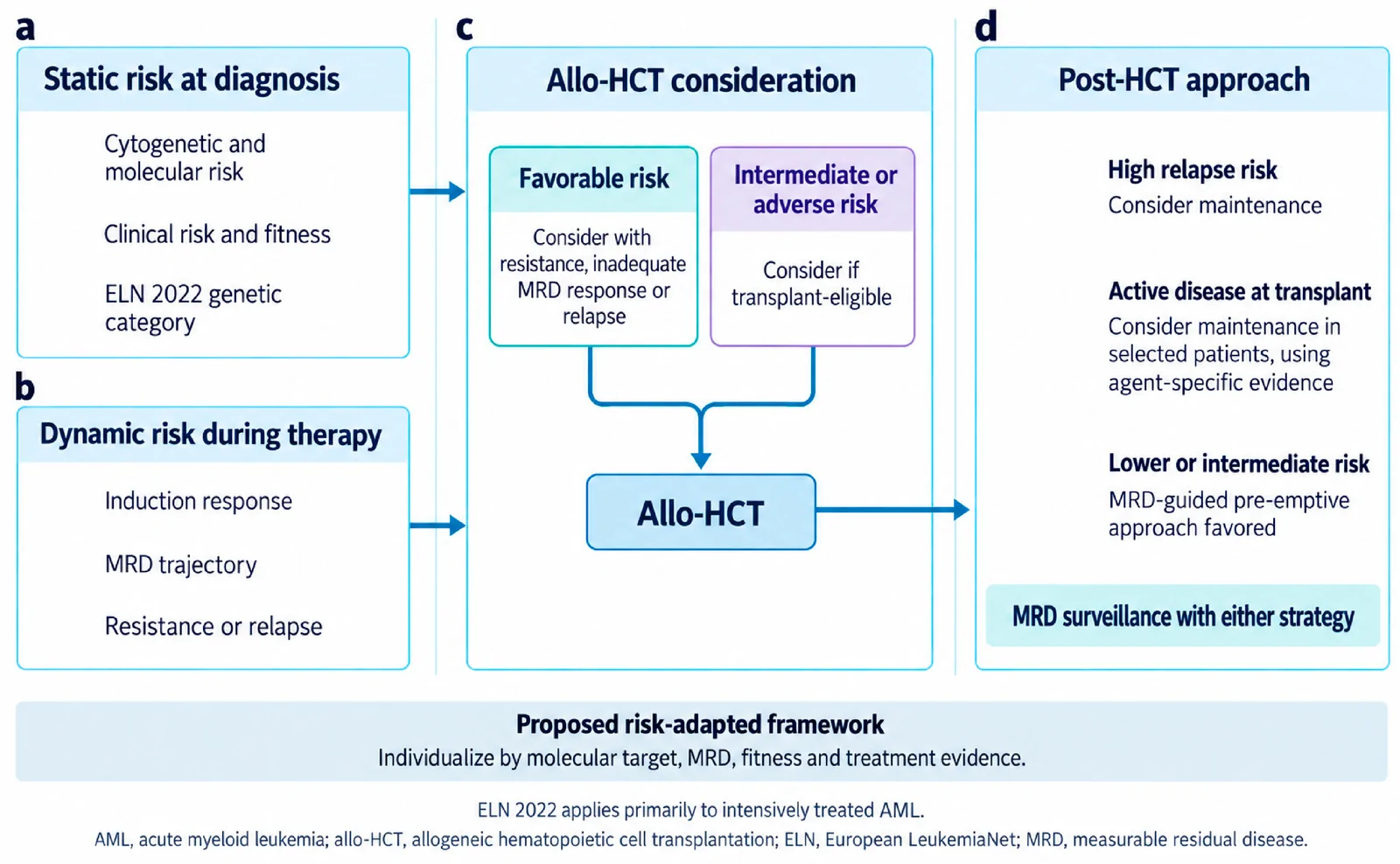
Risk-adapted allo-HCT and post-transplant strategy in AML. The figure summarizes four key concepts: static risk at diagnosis, determined by clinical risk and captured by ELN 2022 categories (**a**); dynamic risk during therapy, defined by response to induction, primary resistance and relapse (**b**); allo-HCT indication according to ELN 2022 risk, with favorable-risk patients generally proceeding to transplant in case of resistance, inadequate MRD response or relapse and intermediate/adverse-risk patients considered for transplant if eligible (**c**); and post-HCT intervention, with maintenance proposed for high-risk patients and considered for selected patients transplanted with active disease, according to agent-specific evidence, whereas low/favorable- and intermediate-risk patients are preferentially managed with MRD-guided pre-emptive strategies. MRD surveillance remains appropriate during both maintenance and pre-emptive strategies (**d**).

**Figure 3:**
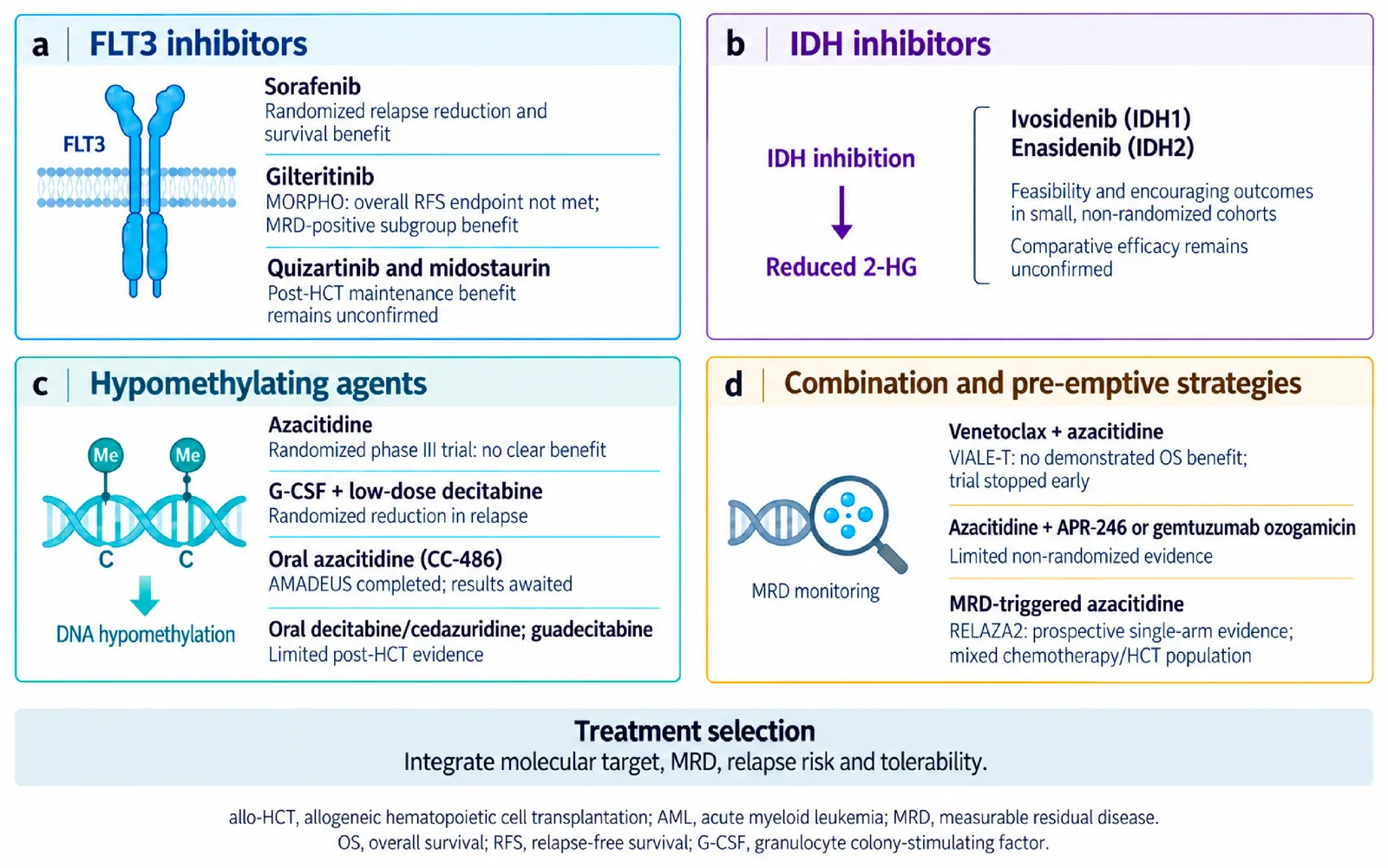
Drug evidence overview. Conceptual overview of pharmacologic maintenance and pre-emptive strategies after allogeneic hematopoietic stem cell transplantation in acute myeloid leukemia. The figure organizes currently investigated approaches according to therapeutic class, biological rationale and maturity of clinical evidence. FLT3 inhibitors constitute the best-developed mutation-directed strategy, with sorafenib supported by the most consistent post-transplant data and gilteritinib suggesting an MRD-adapted role in patients with detectable peri-transplant disease (**a**). IDH1/2 inhibitors provide a rational mutation-specific approach but remain supported mainly by early-phase feasibility studies (**b**). Hypomethylating agents, including azacitidine, decitabine, oral azacitidine, decitabine/cedazuridine and guadecitabine, offer broader applicability through epigenetic and immunomodulatory mechanisms, although randomized evidence has been inconsistent and treatment deliverability remains a major limitation (**c**). Combination regimens and MRD-triggered pre-emptive approaches are promising but require prospective validation to define optimal patient selection, timing, duration and integration with graft-versus-leukemia effects (**d**).

**Table 1:** Summary of FLT3 inhibitor maintenance post-transplant.

Agent	Evidence Base	Main Finding	Clinical Interpretation
Sorafenib	Retrospective studies, phase I feasibility data, SORMAIN, and a randomized phase III trial.	Reduced post-transplant relapse and improved survival outcomes in FLT3-ITD-mutated AML.	Best-established FLT3 inhibitor for post-allo-HCT maintenance; use should be individualized according to tolerability, GVHD, drug interactions and treatment duration [24]
Gilteritinib	MORPHO, a randomized, double-blind, placebo-controlled phase III trial of 24-month post-transplant maintenance.	No significant RFS benefit in the overall population, but improved RFS in patients with detectable peri-transplant FLT3-ITD MRD.	Supports an MRD-adapted maintenance strategy, particularly in patients with persistent or emerging molecular disease [27]. Current ELN-DAVID guidance specifies FLT3-ITD MRD detected by an ultrasensitive molecular assay [15]
Quizartinib	QuANTUM-First exploratory maintenance analyses and limited post-transplant data from QuANTUM-R.	In QuANTUM-First, quizartinib improved OS in newly diagnosed FLT3-ITD AML overall, but an OS benefit of quizartinib maintenance was not demonstrated among transplanted patients; in QuANTUM-R, limited numbers suggested longer OS in patients receiving post-transplant quizartinib maintenance.	Promising but not yet established as post-allo-HCT maintenance; interpretation is limited by exploratory analyses and small post-transplant cohorts. Patients were not re-randomized before maintenance [30]
Midostaurin	RADIUS, a randomized open-label phase II exploratory trial of standard of care with or without midostaurin after allo-HCT.	Estimated 18-month RFS was numerically higher with midostaurin plus standard of care than with standard of care alone, without statistical significance; greater in vivo FLT3 inhibition was associated with improved RFS.	Feasible but supported by limited evidence; tolerability and dose modifications may restrict its use, and stronger randomized data are needed before routine post-transplant maintenance adoption [29]

**Table 2:** Summary of IDH inhibitor maintenance after transplant.

Agent	Evidence Base	Main Finding	Clinical Interpretation
Enasidenib	Phase I post-allo-HCT maintenance study in IDH2-mutated AML using dose escalation; treatment started between days 30 and 90 and continued for up to twelve 28-day cycles. Additional pilot and phase II cohorts treated 15 and 47 patients, respectively	RP2D was 100 mg once daily; grade ≥ 3 toxicities were uncommon; relapse incidence was 16%; 2-year PFS and OS were 69% and 74%, respectively. Completion varied: 12/15 completed 24 cycles in the pilot and 24/47 completed 12 cycles in IDH2 post-alloHCT	Feasible and generally well tolerated, with preliminary antileukemic activity; efficacy remains unproven because data are early-phase, non-randomized and based on small cohorts.
Ivosidenib	Multicenter phase I post-allo-HCT maintenance trial in IDH1-mutated AML using dose de-escalation; treatment started between days 30 and 90 and continued for up to twelve 28-day cycles.	RP2D was 500 mg once daily; severe treatment-related adverse events were infrequent; 2-year relapse and non-relapse mortality were 19% and 0%; 2-year PFS and OS were 81% and 88%.	Promising safety and survival signals support further study, but current evidence is insufficient for routine use outside clinical trials.
Overall IDH inhibitor maintenance	Evidence derives from early-phase feasibility and safety studies without randomized comparisons.	Oral, mutation-directed agents appear compatible with prolonged use and may reduce relapse risk, but definitive efficacy has not been established.	Investigational strategy; future studies should clarify patient selection, duration, MRD-guided intervention and interaction with graft-versus-leukemia effects.

**Table 3:** Summary of hypomethylating agent maintenance.

Agent or Strategy	Evidence Base	Main Finding	Clinical Interpretation
Azacitidine	Phase I studies, immune-modulation studies, and the largest randomized trial versus observation in high-risk AML/MDS after allo-HCT.	Feasible at low doses, with evidence of immune modulation; however, randomized data did not show significant improvement in RFS or OS; treatment exposure was limited.	Biologically plausible but not established as standard maintenance; its use remains limited by deliverability, cytopenias, infections, and uncertain patient selection.
Decitabine	Small phase I dose-escalation studies and post-transplant feasibility cohorts.	Low-dose administration was feasible and did not clearly exacerbate GVHD, but hematologic toxicity was frequent and comparative efficacy remains unproven.	Investigational strategy; further controlled studies are required to define dose, duration, and the patients most likely to benefit.
G-CSF plus low-dose decitabine	Open-label randomized phase II study in MRD-negative AML patients after transplantation.	Associated with lower 2-year relapse incidence than observation, without an apparent increase in chronic GVHD; immune-cell expansion suggested a possible immunomodulatory effect.	Promising but not definitive; findings need validation and may not be generalizable beyond selected MRD-negative patients.
Oral azacitidine (CC-486)	Phase I/II post-allo-HCT study and completed randomized phase III evaluation, with results not posted as of September 2026.	Feasible in selected patients and generally feasible and better aligned with prolonged exposure, with a possible relapse-control signal in small non-randomized cohorts.; grade 3–4 treatment-emergent adverse events occurred in 22/30, and 13/30 completed 12 cycles.	Potentially attractive for long-term maintenance, but efficacy remains unconfirmed pending randomized results.
Oral decitabine/cedazuridine	Approved in MDS outside the post-transplant setting; post-allo-HCT evidence includes a retrospective MDS cohort and the single-arm GFM-DACORAL-DLI trial; additional studies are ongoing.	Oral administration may improve feasibility and support sustained epigenetic exposure but comparative efficacy remains undefined; GFM-DACORAL-DLI reported substantial hematologic toxicity and included concomitant DLI.	Experimental in the maintenance setting; appropriate mainly within prospective studies.
Guadecitabine	Single-center phase II study across maintenance, pre-emptive, and salvage post-transplant settings.	Showed acceptable safety and preliminary activity, including MRD eradication in a subset; however, ASTRAL studies were negative, and the post-transplant investigators reported discontinued drug supply.	Clinically informative but unlikely to become a practical maintenance option because of discontinued development and lack of confirmatory evidence.
Overall HMA maintenance	Evidence includes small prospective studies, one negative randomized azacitidine trial, and heterogeneous meta-analytic data.	HMAs may reduce relapse in selected contexts, but results are inconsistent and strongly influenced by treatment exposure, patient selection, MRD integration, and study design.	HMA maintenance remains investigational rather than broadly standard; future implementation should be biologically informed, MRD-adapted, and supported by prospective controlled trials.

**Table 4:** Active/recruiting clinical trials on maintenance therapy after allo-HCT (from ClinicalTrials.gov).

NCT Number	Intervention	Trial Status—Year	Phase
NCT07304232	Chidamide post-allo-HCT in CR—AML	Recruiting—2026	II
NCT05270200	Chidamide plus azacitidine post-allo-HCT in high risk AML	Recruiting—2026	I/II
NCT06575296	Revumenib post-allo-HCT in high risk AML	Recruiting—2026	I
NCT06440135	Ziftomenib post-allo-HCT in high risk AML	Recruiting—2026	I
NCT07686965	Lisaftoclax plus azacitidine post-allo-HCT in high risk AML	Recruiting—2026	III
NCT03932643	ONC201 post-allo-HCT in high risk AML	Recruiting—2026	I
NCT06543381	Olutasidenib after allo-HCT in IDH1 AML	Recruiting—2026	I

AML = acute myeloid leukemia; allo-HCT = allogeneic stem cell transplant; CR = complete remission.

## Data Availability

No new data were created or analyzed in this study. Data sharing is not applicable to this article.
